# Supplementary material for: Construction of Brain Metastasis Prediction Model and Optimization of Prophylactic Cranial Irradiation Selection for Limited-Stage Small-Cell Lung Cancer
Source: Cancers (Basel). 2022 Oct 7;14(19):4906. doi: 10.3390/cancers14194906 (PMC9563012; doi:10.3390/cancers14194906)
Supplement: Supplementary file 1 [file cancers-14-04906-s001.zip › cancers-1909661-supplementary.pdf]

Table S1 Clinical characteristics of patients in the training cohort and validation cohort

Variable	Cutoff	Statistic	ALL patients	Training Cohort	Validation Cohort	*p*
Clinical stage						1.000
I-II			17(10.1%)	12(9.92%)	5(10.64%)	
III			151(89.9%)	109(90.08%)	42(89.36%)	
Gender						0.165
Male			37(22%)	30(24.79%)	7(14.89%)	
Female			131(78%)	91(75.21%)	40(85.11%)	
Age						0.421
<70			81(48.2%)	56(46.28%)	25(53.19%)	
≥70			87(51.8%)	65(53.72%)	22(46.81%)	
ECOG						0.835
0-1			141(83.9%)	102(84.3%)	39(82.98%)	
2-4			27(16.1%)	19(15.7%)	8(17.02%)	
Smoke						0.269
Yes			46(27.4%)	36(29.75%)	10(21.28%)	
No			122(72.6%)	85(70.25%)	37(78.72%)	
BMI						0.984
Normal			111(66.1%)	80(66.12%)	31(65.96%)	
Abnormal			57(33.9%)	41(33.88%)	16(34.04%)	
CT cycles						0.33
<4			21(12.5%)	17(14.05%)	4(8.51%)	
≥4			147(87.5%)	104(85.95%)	43(91.49%)	
Time To RT	1.8	2.79				1.000
≥1.8			96(57.1%)	69(57.02%)	27(57.45%)	
<1.8			55(32.7%)	40(33.06%)	15(31.91%)	
No RT			17(10.1%)	12(9.92%)	5(10.64%)	
HGB						0.763
Normal			15(8.9%)	10(8.26%)	5(10.64%)	
Abnormal			153(91.1%)	111(91.74%)	42(89.36%)	
Na						0.370
Normal			136(81%)	100(82.64%)	36(76.6%)	
Abnormal			32(19%)	21(17.36%)	11(23.4%)	
LYM	2.37	1.104813				0.337
Low			139(82.7%)	98(80.99%)	41(87.23%)	
High			29(17.3%)	23(19.01%)	6(12.77%)	
PLT	200	1.726967				0.939
Low			28(16.7%)	20(16.53%)	8(17.02%)	
High			140(83.3%)	101(83.47%)	39(82.98%)	
MPV	10.4	1.150909				0.866
Low			52(31%)	37(30.58%)	15(31.91%)	
High			116(69%)	84(69.42%)	32(68.09%)	
LDH	206	2.598067				0.367
Low			80(47.6%)	55(45.45%)	25(53.19%)	
High			88(52.4%)	66(54.55%)	22(46.81%)	
AGR	1.74	2.022924				0.118
Low			125(74.4%)	94(77.69%)	31(65.96%)	
High			43(25.6%)	27(22.31%)	16(34.04%)	
UA	173	2.196544				0.948
Low			21(12.5%)	15(12.4%)	6(12.77%)	

High			147(87.5%)	106(87.6%)	41(87.23%)	
CysC	0.75	1.695112				0.186
Low			55(32.7%)	36(29.75%)	19(40.43%)	
High			113(67.3%)	85(70.25%)	28(59.57%)	
CEA	2.22	1.369027				0.622
Low			95(56.5%)	67(55.37%)	28(59.57%)	
High			73(43.5%)	54(44.63%)	19(40.43%)	
NSE	25.22	3.1664				0.777
Low			145(86.3%)	105(86.78%)	40(85.11%)	
High			23(13.7%)	16(13.22%)	7(14.89%)	
ProGRP	1740	2.759637				0.145
Low			149(88.7%)	110(90.91%)	39(82.98%)	
High			19(11.3%)	11(9.09%)	8(17.02%)	
CA125	27.72	1.790778				0.738
Low			133(79.2%)	95(78.51%)	38(80.85%)	
High			35(20.8%)	26(21.49%)	9(19.15%)	
NLR	1.4852941	2.107099				0.473
Low			23(13.7%)	18(14.88%)	5(10.64%)	
High			145(86.3%)	103(85.12%)	42(89.36%)	
PLR	252.4752475	2.303172				0.73
Low			142(84.5%)	103(85.12%)	39(82.98%)	
High			26(15.5%)	18(14.88%)	8(17.02%)	
ALI	699.2275877	1.536799				0.397
Low			148(88.1%)	105(86.78%)	43(91.49%)	
High			20(11.9%)	16(13.22%)	4(8.51%)	
SIIRI	2.3945161	1.740514				0.613
Low			139(82.7%)	99(81.82%)	40(85.11%)	
High			29(17.3%)	22(18.18%)	7(14.89%)	
AAPR	0.8016949	1.112769				0.565
Low			150(89.3%)	107(88.43%)	43(91.49%)	
High			18(10.7%)	14(11.57%)	4(8.51%)	
PNI	38.4081	1.900958				0.545
Low			26(15.5%)	20(16.53%)	6(12.77%)	
High			142(84.5%)	101(83.47%)	41(87.23%)	
LMR	2.6986301	2.565036				0.851
Low			41(24.4%)	30(24.79%)	11(23.4%)	
High			127(75.6%)	91(75.21%)	36(76.6%)	

CT cycles chemotherapy cycles, Time To RT time to chemotherapy to radiotherapy, BMI body mass index, HGB hemoglobin, LYM lymphocyte, PLT platelet, MPV men platelet volume, LDH lactate dehydrogenase, AGR albumin-to-globulin ratio, UA uric acid, CysC cystatin C, CEA carcinoembryonic antigen, NSE neuron-specific enolase, ProGRP pro-gastrin-releasing peptide precursor, CA125 carbohydrate antigen 125, NLR neutrophil-to-lymphocyte ratio, PLR platelet-to-lymphocyte ratio, ALI advanced lung cancer inflammation index, SIRI systemic inflammation response index, AAPR albumin-to-alkaline phosphatase ratio, PNI prognostic nutrition index, LMR lymphocytes monocytes ratio.

Table S2 Univariate analyses for TTBM of LS-SCLC patients

**Variable**	**HR（95%CI）**	***p***
Clinical stage（I-II vs. III）	0.254(0.080-0.808)	0.020
Gender（Male vs Female）	0.686(0.388-1.215)	0.197
Age（≥70 vs <70）	1.261(0.791-2.010)	0.330
ECOG（2-4 vs 0-1）	1.884(1.044-3.400)	0.035
Smoke（Yes vs. No）	0.689(0.406-1.167)	0.166
CT cycles（<4 vs.≥4）	2.084(1.116-3.891)	0.021
Time To RT		0.044
<1.8 vs ≥1.8	0.322(0.115-0.897)	
No RT vs.≥1.8	0.658(0.392 -1.105)	
BMI（<25 vs.≥25）	1.246(0.767-2.026)	0.374
Na（Normal vs. Abnormal）	1.533(0.851-2.763)	0.155
HGB（Normal vs. Abnormal）	1.035(0.473-2.265)	0.931
NLR（Low vs. High）	1.390(0.746-2.590)	0.300
PLR（Low vs. High）	1.735(0.944-3.189)	0.076
LYM（Low vs. High）	1.279(0.723-2.263)	0.398
PLT（Low vs. High）	0.823(0.432-1.569)	0.554
MPV（Low vs. High）	0.995(0.597-1.659)	0.985
LDH（Low vs. High）	0.625(0.389-1.006)	0.053
AGR（Low vs. High）	0.413(0.205-0.835)	0.014
UA（Low vs. High）	1.522(0.817-2.835)	0.186
CysC（Low vs. High）	0.911(0.538-1.542)	0.727
CEA（Low vs. High）	0.710(0.440-1.148)	0.162
NSE（Low vs. High）	2.220(1.203-4.095)	0.011
ProGRP（Low vs. High）	2.546(1.288-5.034)	0.007
CA125（Low vs. High）	1.825(1.035-3.218)	0.038
ALI（Low vs. High）	1.151(0.589-2.247)	0.681
SIRI（Low vs. High）	1.626(0.915-2.890)	0.097
AAPR（Low vs. High）	0.626(0.299-1.314)	0.216
PNI（Low vs. High）	1.632(0.921-2.893)	0.093
LMR（Low vs. High）	1.812(1.090-3.011)	0.022

CT cycles chemotherapy cycles, Time To RT time to chemotherapy to radiotherapy, BMI body mass index, HGB hemoglobin, LYM lymphocyte, PLT platelet, MPV men platelet volume, LDH lactate dehydrogenase, AGR albumin-to-globulin ratio, UA uric acid, CysC cystatin C, CEA carcinoembryonic antigen, NSE neuron-specific enolase, ProGRP pro-gastrin-releasing peptide precursor, CA125 carbohydrate antigen 125, NLR neutrophil-to-lymphocyte ratio, PLR platelet-to-lymphocyte ratio, ALI advanced lung cancer inflammation index, SIRI systemic inflammation response index, AAPR albumin-to-alkaline phosphatase ratio, PNI prognostic nutrition index, LMR lymphocytes monocytes ratio.
